# White-nose syndrome-related changes to Mid-Atlantic bat communities across an urban-to-rural gradient

**DOI:** 10.1186/s40850-021-00079-5

**Published:** 2021-05-07

**Authors:** Sabrina Deeley, Joshua B. Johnson, W. Mark Ford, J. Edward Gates

**Affiliations:** 1 Department of Fish and Wildlife Conservation, Virginia Tech, Blacksburg, VA 24061 USA; 2 Pennsylvania Game Commission, Harrisburg, PA 17110 USA; 3 U.S. Geological Survey, Virginia Cooperative Fish and Wildlife Research Unit, Blacksburg, VA 24061 USA; 4 University of Maryland Center for Environmental Science, Appalachian Laboratory, Frostburg, MD 21532 USA

**Keywords:** Bat, *Eptesicus fuscus*, *Lasiurus borealis*, *Myotis septentrionalis*, Urban environments, White-nose syndrome

## Abstract

**Background:**

White-nose Syndrome (WNS) has reduced the abundance of many bat species within the United States’ Mid-Atlantic region. To determine changes within the National Park Service National Capital Region (NCR) bat communities, we surveyed the area with mist netting and active acoustic sampling (2016–2018) and compared findings to pre-WNS (2003–2004) data.

**Results:**

The results indicated the continued presence of the threatened *Myotis septentrionalis* (Northern Long-eared bat) and species of conservation concern, including *Perimyotis subflavus* (Tri-colored bat), *Myotis leibii* (Eastern Small-footed bat) and *Myotis lucifugus* (Little Brown bat). However, we documented a significant reduction in the abundance and distribution of *M. lucifugus* and *P. subflavus*, a decrease in the distribution of *M. septentrionalis*, and an increase in the abundance of *Eptesicus fuscus* (Big Brown bat).

**Conclusions:**

Documented post-WNS *M. septentrionalis* recruitment suggests that portions of the NCR may be important bat conservation areas. Decreases in distribution and abundance of *P. subflavus* and *M. lucifugus* indicate probable extirpation from many previously occupied portions of the region.

## Background

White-nose Syndrome is caused by the psychrophilic fungi *Pseudogymnoascus destructans* [1–3], an invasive species native to Eurasia [4–8]. White-nose Syndrome has caused widespread mortality of cave-hibernating bats [9–11]. For hibernating bats, fungal invasion of epithelial tissue increased overwinter arousal, leading to a depletion of fat reserves followed by mortality from starvation or, for overwinter survivors, decreased post-hibernating condition, decreased fitness, and shifts in reproductive timing [9, 12–15]. Since WNS was first observed in New York in 2006, *P*. *destructans* has spread to 33 states in the United States (U.S.) and 7 provinces in Canada, including hibernacula presumably used by bats summering within the national capital region (NCR) around Washington, District of Columbia (D.C.) [16]. Based largely on winter hibernacula counts species, such as *Myotis lucifugus* (Little Brown bat), *Myotis septentrionalis* (Northern Long-eared bat), *Myotis sodalis* (Indiana bat), and *Perimyotis subflavus* (Tri-colored bat), have undergone large population decreases [10, 11, 17]. In the spring, these species migrate from hibernacula to summer habitat [18]. Therefore, summer activity surveys have reflected WNS-caused population declines of *M. lucifugus*, *M. septentrionalis*, and *M. sodalis* in Massachusetts, New Hampshire, New York and West Virginia [12, 14, 19–23].

White-nose Syndrome does not impact all bat species equally. Roosting behaviors, body sizes, hibernacula colony sizes, environmental conditions of particular hibernacula, WNS exposure levels and disease virulence can positively or negatively affect survival rates following exposure to WNS [24, 25]. Although WNS can negatively impact *Eptesicus fuscus* (Big Brown bat) [19], many individuals appear to be unaffected or are able to survive at higher rates than other species, perhaps because their larger body size allows for greater fat reserves than smaller bat species [26], or they are hibernating within warmer anthropogenic roosts unfavorable to growth of *P. destructans* [27]. Despite high mortality rates, remnant populations of *M. lucifugus*, *M. septentrionalis*, *M. sodalis*, and *P. subflavus* can persist in WNS-affected areas [28–30].

When populations experience declines or extirpation, competitive release may result in other species altering their habitat use, increasing their range, or increasing their population size; for example, some relaxation in niche partitioning has been observed for bats in WNS-impacted landscapes [31]. Along the East Coast, increases in *E. fuscus*, *Lasiurus borealis* (Eastern Red bat), and *Lasiurus cinereus* (Hoary bat) have corresponded with decreases in *M. lucifugus*, *M. septentrionalis*, *M. sodalis* and *P. subflavus* [12, 19, 31]. However, total intra-night activity indicated that other bat species were not expanding into temporal niches formerly dominated by *M. lucifugus* post-WNS [23, 31].

Urban environments can increase contact between individuals because of highly-concentrated resources, such as limited foraging patches and limited day-roost availability, or impact individuals’ physical condition through use of marginal day roosts, increased competition for food resources, and pollution [32]. Urbanization can impact bat activity patterns and foraging ecology [33]. Therefore, urbanization may exacerbate winter WNS population declines, as better body condition entering hibernation can increase WNS survival [34, 35] or compound WNS-caused population declines with urbanization-caused decreases in reproductive success [12, 15, 29, 36]. Additionally, highly-urbanized areas often have lower bat diversity and activity levels than less-developed, rural, semi-natural, or natural areas [36–43].

Hibernacula near the NCR have shown large declines in *M. lucifugus*, *M. sodalis*, and *P. subflavus* populations [17, 44]. Accordingly, we sought to document how summer bat communities and habitat associations in the NCR have changed since WNS onset, in terms of overall bat activity and changes to community membership. In 2003–2004, Johnson et al. [45] surveyed bat communities within 11 NCR National Park Service (NPS) units to assess community patterns along an urban-to-rural gradient using a combined active acoustic and mist-netting sample approach. Both methods allow for detection of bat species and evaluation of relative activity or abundance. Acoustic sampling records bat echolocation calls that can be identified to species, and mist netting captures and identifies individual bats [46].

Our first objective was to compare the sampling effort required to document presence of species pre- and post-WNS. We expected that the effort required to document the same species would be higher post-WNS than pre-WNS [46]. Our second objective was to determine the impacts of WNS on bat communities within the NCR by considering relative abundance measures and distribution of detections in the NCR. We predicted shifts in NCR community structure relative to the pre-WNS findings of Johnson et al. [45], including possible population decreases, population extirpations, and reduced distribution of species highly impacted by WNS (*M. lucifugus*, *M. septentrionalis*, *M. sodalis*, *and P. subflavus*). Competitive release of larger-bodied, less-affected species, such as *E. fuscus,* or non-hibernating migratory species, such as *L. borealis,* could result in increased abundance or changes in habitat associations. Similar to Johnson et al. [45], we predicted that the highest species richness would be found in less-fragmented, rural habitats with more forest cover, as fewer bat species occurred within higher urbanization levels.

## Methods

### Study area

**Fig. 1:**
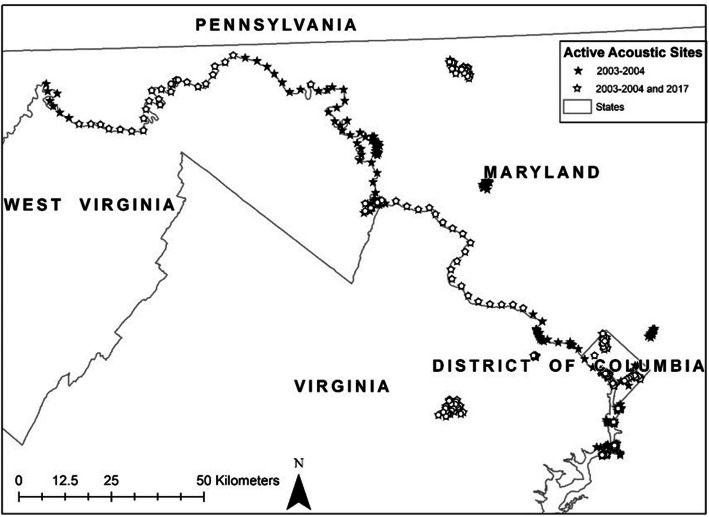
National Park Service National Capital Region acoustic survey sites during pre-White-nose Syndrome (2003–2004) and post-White-nose Syndrome (2016–2018) periods. Black stars indicate sites surveyed 2003–2004, and open stars indicate sites surveyed in both 2003–2004 and 2017. Park abbreviations are: Antietam National Battlefield (ANTI), Catoctin Mountain Park (CATO), Chesapeake and Ohio Canal National Historical Park (CHOH), George Washington Memorial Parkway (GWMP), Harpers Ferry National Historical Park (HAFE), Manassas National Battlefield Park (MANA), Monocacy National Battlefield (MONO), National Capital Parks-Central [now National Mall and Memorial Parks (NAMA)], National Capital Parks-East (NACE), Rock Creek Park (ROCR), and Wolf Trap National Park for the Performing Arts (WOTR). CHOH follows the Maryland border with Virginia and West Virginia from the western-most part of the study area to downtown District of Columbia (D.C.) between the two CHOH points. NACE and GWMP points indicate the southern-most and northern-most parks within those units. Parks partially or entirely within D.C. include ROCR, CHOH, NAMA, and NACE

Our study area consisted of the 11 NPS units that Johnson et al. [45] surveyed 2003–2004: Antietam National Battlefield (ANTI), Catoctin Mountain Park (CATO), Chesapeake and Ohio Canal National Historical Park (CHOH), George Washington Memorial Parkway (GWMP), Harpers Ferry National Historical Park (HAFE), Manassas National Battlefield Park (MANA), Monocacy National Battlefield (MONO), National Capital Parks-Central (now National Mall and Memorial Parks [NAMA]), National Capital Parks-East (NACE), Rock Creek Park (ROCR) and Wolf Trap National Park for the Performing Arts (WOTR; Fig. 1). The area covers portions of the Coastal Plain (CHOH, GWMP, NACE, NAMA), Piedmont (CHOH, GWMP, MANA, MONO, ROCR, WOTR), Blue Ridge Mountains (CATO, CHOH, HAFE) and Ridge and Valley (ANTI, CHOH, HAFE) physiographic regions in D.C., Maryland, Virginia, and West Virginia. Elevations in the sampled sites ranged from sea level to 730 m, with higher elevations and more complex topography in the western Blue Ridge and Ridge and Valley regions. From May to August 2003–2004 and 2016–2018, temperatures during sampling ranged from minima of 2.1–25.4 °C and maxima of 14.6–35.6 °C. Precipitation ranged from 290 to 1157 mm per season, but was highly variable among years, with 2018 receiving more precipitation than other years. White-nose Syndrome was first confirmed in the area over the 2009–2010 winter period [16].

The region includes forests, pasture areas, suburban development, and high-density urban development. The study area largely has an east-west (and, to a lesser extent, south-north) urban-to-rural gradient. The most rural portions of the study area include CATO (a contiguous forest unit surrounded by both private and public forestland) and northwestern portions of CHOH in central and western Maryland. As the longest unit with the most habitat variation, CHOH follows the D.C. and Maryland border with Virginia and West Virginia from downtown D.C., through suburban development, agricultural fields, and private and state forests and wildlife management areas to Cumberland, Maryland. The CHOH is adjacent to HAFE (a mix of rural mountainous forest and open battlefields), near ANTI and MONO, and adjacent to small portions of GWMP. The battlefield units (ANTI, MANA, MONO) consist mainly of agricultural fields and small forest woodlots. As a performing arts center, WOTR is a combination of developed land and forest fragments surrounded by a developed suburban landscape. Near D.C., GWMP primarily follows the Virginia border and Potomac River. It contains larger contiguous forest stands (e.g., Great Falls Park, approximately 13 km north of D.C.) and linear forest strips along motorways. Units partially or entirely within D.C. (ROCR, CHOH, NAMA, NACE) are highly fragmented, with the exception of ROCR. This unit primarily consists of 2 large contiguous forested patches which follow 2 water courses to the Potomac River, Rock Creek and Foundry Branch [47]. Dominated by open areas and National Mall monuments, NAMA contains minimal forest cover. The parks of NACE include units on the southeastern bank of the Anacostia River, as well as a thin linear forest strip running along the Baltimore-Washington Parkway up to Greenbelt Park, a small contiguous forest stand northeast of the city.

Currently, the region is dominated by a mix of oak-hickory and mesophytic forest types, e.g., *Quercus* spp. (oak species), *Acer* spp. (maple species), *Liriodendron tulipifera* (yellow poplar) and to a lesser extent *Pinus* spp. (pine species) [48]. Within the NCR units, forest stands primarily consisted of mature and late successional forests, more so than the surrounding landscape [47]. Deciduous tree species that dominate the upland areas on NCR units include *Acer rubrum* (Red Maple), *Fagus grandifolia* (American Beech), *L. tulipifera*, and *Quercus* spp. Riparian forests along larger watercourses contain *Platanus occidentalis* (Sycamore), *Acer negundo* (Boxelder), and *Liquidambar styraciflua* (Sweetgum).

### Acoustic and mist-netting surveys

We replicated the active acoustic methods used by Johnson et al. [45] to allow a direct comparison of echolocation call data between 2003 and 2004 (pre-WNS) and 2017 (post-WNS). Using the same acoustic recording methods was important for directly comparing pre-WNS call data recorded to post-WNS call data, as the methods can impact detection and habitat associations [49]. We used Anabat II (Titley Electronics, Ballina, Australia) zero-crossing, frequency-division equipment to actively survey at each sample site for one 20-min period between sunset and 0200. If a bat was “observed” as evidenced by audible echolocation sounds from the recorder or by sight, we attempted to follow its direction with the microphone to record a high-quality call sequence [50]. Active survey sites were in forested, wetland, and open habitats with a mean distance between survey locations of 886 m ± 982 m [45]. We avoided active acoustic recording and netting during heavy precipitation or low temperatures, as these can reduce bat activity [51].

In 2003 and 2004, we surveyed 362 sites, and in 2017, we revisited 147 sites (40.6%; Fig. 1). All echolocation calls (2003–2004 and 2017) were identified to species using the U.S Fish and Wildlife Service approved Kaleidoscope 4.5.0 Bats of North America 4.2.0 classifier (Wildlife Acoustics, Inc., Concord, MA, USA) with the following species considered as known or possibly extant in the NCR: *E. fuscus*, *Lasiurus borealis*, *Lasiurus cinereus*, *Lasionycteris noctivagans*, *Myotis leibii* (Eastern Small-footed bat), *M. lucifugus*, *M. septentrionalis*, *M. sodalis*, *Nycticeius humeralis* (Evening bat) and *P. subflavus*. We used the number of echolocation calls identified to species as response variables in statistical analyses.

We mist-netted from 28 May – 29 August in 2003 and 2004 (pre-WNS), as outlined in Johnson et al. [45]. Between 20 April and 10 August 2016–2018 (post-WNS), we conducted mist-netting in these same NPS units, and in some cases in the same survey locations. Most post-WNS sampling effort was 15 May – 15 August, per the Range-wide Indiana Bat Survey Guidelines [52]. Post-WNS, netting locations were selected both to provide a general survey of the region and additional effort in areas with acoustic and mist-netting detections of *M. lucifugus*, *M. septentrionalis*, *M. sodalis* and *P. subflavus* [43, 53]. Because of post-WNS captures of *M. septentrionalis* at ROCR [53], we allocated additional effort there in 2018. We placed mist nets along possible flight corridors, e.g., trails, roads with forest canopy cover, and/or near water. The number and size of nets varied by forest structure and logistics. Bat capture and handling procedures were approved under Virginia Tech Institutional Animal Care and Use protocol 16–049 and allowed under USFWS #TE34778A-2, Maryland Department of Natural Resources #56152, Virginia Department of Game and Inland Fisheries #061519, and West Virginia Division of Natural Resources #2018.180 scientific collecting permits*.* We identified bats to species using a dichotomous key to the bats of this region [54], and recorded sex, age (adult or juvenile), and reproductive condition [55, 56]. We disregarded samples when heavy precipitation or fog event (as defined by on-the-ground observations) disrupted sampling.

### Data analysis

We developed species accumulation curves for both our acoustic and netting datasets, which provided an assessment of species richness based on the level of sample effort [57]. We developed species accumulation curves for each pre- or post-WNS dataset using the R package *vegan* with random selection [58]. In the two acoustic species-accumulation curves, we used all samples and 10 acoustically-detected species. For the pre- and post-WNS species accumulation curves based on mist-net sampling, we selected the nights with at least 4 h of netting and no precipitation within the netting event, based on on-the-ground observations. We created 2 sets of species accumulation curves for the netting data: 1 with the 6 species that were found both pre- and post-WNS and 1 for all 9 species captured post-WNS.

To determine whether the number of echolocation calls and number of bat captures for each species changed based on WNS, we performed paired and unpaired *t*-tests in program R [59–61]. We used paired *t*-tests for all acoustic sites that were sampled both pre- and post-WNS. We used unpaired t-tests for analysis of all netting sample data because we did not resample the exact same locations pre- and post-WNS. The netting *t*-tests used nightly captures per net to account for higher netting efforts post-WNS.

## Results

We recorded 4967 echolocation calls (2003–2004 = 3637; 2017 = 1330), which were identified to 10 species (Table 1). Species richness declined in every NPS unit except NACE, with all units losing detections of at least 1 species severely impacted by WNS (Table 2). Pre-WNS survey years had higher proportions of nights with identified bat echolocation calls (2003 = 80.0%, 2004 = 82.9%) than the post-WNS sample year (2017 = 69.4%; Table 1).

**Table 1:** The total number of active acoustic survey samples with presence of each species (Sites), total bat echolocation calls (Total), and mean and standard error (SE) of bat echolocation calls per active acoustic sample. Samples were collected 2003–2004 and 2017 in the National Park Service National Capital Region. Mean change is the 2017 mean minus the 2003–2004 mean. *P*-values were from Pearson’s paired *t*-test. Calls were identified to species by using Kaleidoscope 4.5.0 Bats of North America 4.2.0 classifier (Wildlife Acoustics Inc., Concord, MA, USA) for the following species: *Eptesicus fuscus* (Big Brown bat; EPFU), *Lasiurus borealis* (Eastern Red bat; LABO), *Lasiurus cinereus* (Hoary bat; LACI), *Lasionycteris noctivagans* (Silver-haired bat; LANO), *Myotis leibii* (Eastern Small-footed bat; MYLE), *Myotis lucifugus* (Little Brown bat; MYLU), *Myotis septentrionalis* (Northern-Long eared bat; MYSE), *Myotis sodalis* (Indiana bat; MYSO), *Nycticeius humeralis* (Evening bat; NYHU) and *Perimyotis subflavus* (Tri-colored bat; PESU)

Species code	2003–2004			2017			Mean change	*P*-value
	Sites	Total	Mean (SE)	Sites	Total	Mean (SE)		
EPFU	139	889	2.46 (0.40)	60	679	4.62 (0.96)	2.16	0.01
LABO	77	151	0.42 (0.06)	39	104	0.71 (0.17)	0.29	0.07
LACI	125	496	1.37 (0.21)	35	85	0.58 (0.13)	−0.79	0.07
LANO	150	878	2.43 (0.34)	55	251	1.71 (0.32)	−0.72	0.86
MYLE	17	23	0.06 (0.02)	1	1	0.01 (0.01)	−0.05	0.05
MYLU	106	573	1.58 (0.33)	29	66	0.44 (0.09)	−1.14	0.00
MYSE	28	48	0.13 (0.03)	1	13	0.09 (0.08)	−0.04	0.33
MYSO	28	77	0.21 (0.05)	8	9	0.06 (0.02)	−0.15	0.01
NYHU	54	108	0.30 (0.52)	34	94	0.64 (0.18)	0.34	0.13
PESU	81	394	1.09 (0.21)	14	28	0.19 (0.07)	−0.90	0.01

**Table 2:** Species richness documented through active acoustic and mist-net sampling in National Park Service National Capital Region units 2003–2004 and 2016–2018. Species lost indicate species that were documented 2003–2004 but not 2016–2018. Species gained are those species documented in 2016–2018 but not 2003–2004. Note that sample effort by unit was not equal between 2003 and 2004 and 2016–2018. Unit abbreviations are: Antietam National Battlefield (ANTI), Catoctin Mountain Park (CATO), Chesapeake and Ohio Canal National Historical Park (CHOH), George Washington Memorial Parkway (GWMP), Harpers Ferry National Historical Park (HAFE), Manassas National Battlefield Park (MANA), Monocacy National Battlefield (MONO), National Capital Parks-Central [now National Mall and Memorial Parks (NAMA)], National Capital Parks-East (NACE), Rock Creek Park (ROCR), and Wolf Trap National Park for the Performing Arts (WOTR). Species abbreviations are: *Eptesicus fuscus* (Big Brown bat; EPFU), *Lasiurus borealis* (Eastern Red bat; LABO), *Lasiurus cinereus* (Hoary bat; LACI), *Lasionycteris noctivagans* (Silver-haired bat; LANO), *Myotis leibii* (Eastern Small-footed bat; MYLE), *Myotis lucifugus* (Little Brown bat; MYLU), *Myotis septentrionalis* (Northern Long-eared bat; MYSE), *Nycticeius humeralis* (Evening bat; NYHU) and *Perimyotis subflavus* (Tri-colored bat; PESU)

Sample method	Unit	Species richness		Species lost	Species gained
		2003–2004	2016–2018		
Acoustics	ANTI	9	N/A	N/A	N/A
	CATO	10	8	MYLE, MYSE	
	CHOH	10	8	MYLE, MYSE	
	GWMP	10	N/A	N/A	N/A
	HAFE	8	2	EPFU, LACI, LANO, MYLU, MYSO, PESU	
	MANA	10	8	MYSE, MYSO	
	MONO	7	N/A	N/A	N/A
	NAMA	6	5	PESU	
	NACE	8	8	MYSO	MYSE
	ROCR	8	7	MYSE	
	WOTR	8	1	EPFU, LABO, LANO, MYLU, MYSE, NYHU, PESU	
Netting	ANTI	3	2	MYLU	
	CATO	4	3	MYLU	
	CHOH	6	7	MYSE, LACI	MYLE, NYHU, LANO
	GWMP	4	3	MYLU, PESU	NYHU
	HAFE	3	3	MYSE	LACI
	MANA	3	2	PESU	
	MONO	4	2	MYLU, PESU	
	NAMA	1	1	LABO	EPFU
	NACE	2	2		
	ROCR	6	4	MYLU, PESU, LACI	LANO
	WOTR	2	1	LABO	

Mist-net sampling varied greatly among years: there were more post-WNS than pre-WNS capture events (2003 = 34, 2004 = 40, 2016 = 13, 2017 = 50, 2018 = 98) and later years had higher proportions of nights with bat captures (2003 = 61.8%, 2004 = 85.0%, 2016 = 76.9%, 2017 = 86.0%, 2018 = 92.5%). In 2017 and 2018, netting often had 33–100% more nets per night than pre-WNS surveys, and these years focused on areas where previous passive acoustic surveys had documented presence of species highly impacted by WNS [53], likely increasing the likelihood of capture of rare species. Six species were captured both pre- and post-WNS, and only in post-WNS years, we captured *Lasionycteris noctivagans, M. leibii*, and *N. humeralis* (Tables 2 and 3). Post-WNS, we captured *M. lucifugus* and *P. subflavus* only in northwestern Maryland (CHOH), and their absences from all other units were the primary drivers to post-WNS decreases in unit-level species richness for both netting and acoustics data (Table 2). We noted no evidence of reproduction in the *M. lucifugus* and *P. subflavus* captures, and we only documented evidence of reproduction (pregnant and lactating females) and successful recruitment (juvenile bats) in *M. septentrionalis* populations within ROCR.

**Table 3:** The total number of each species captured (Total Captures), total per mist-net (Total), and mean and standard error (SE) of bat captures per mist-net 2003–2004 and 2016–2018 in the National Park Service National Capital Region. Mean change is the 2016–2018 mean minus the 2003–2004 mean of each species. *P*-values are from Pearson’s *t*-test. Bat species are: *Eptesicus fuscus* (Big Brown bat; EPFU), *Lasiurus borealis* (Eastern Red bat; LABO), *Lasiurus cinereus* (Hoary bat; LACI), *Lasionycteris noctivagans* (Silver-haired bat; LANO), *Myotis leibii* (Eastern Small-footed bat; MYLE), *Myotis lucifugus* (Little Brown bat; MYLU), *Myotis septentrionalis* (Northern Long-eared bat; MYSE), *Nycticeius humeralis* (Evening bat; NYHU) and *Perimyotis subflavus* (Tri-colored bat; PESU)

Species code	Total captures	2003–2004		2016–2018		Mean change	*P*-value
		Total	Mean (SE)	Total	Mean (SE)		
EPFU	958	39.73	0.54 (0.10)	165.77	1.03 (0.11)	0.49	0.01
LABO	181	19.95	0.27 (0.06)	24.01	0.15 (0.02)	−0.12	0.20
LACI	4	0.92	0.01 (0.01)	0.14	0.00 (0.00)	−0.01	0.25
LANO	13	0.00	0.00 (0.00)	2.40	0.01 (0.01)	0.01	0.01
MYLE	1	0	0.00 (0.00)	0.17	0.00 (0.00)	0.00	0.32
MYLU	108	33.8	0.46 (0.17)	0.47	0.00 (0.00)	−0.46	0.01
MYSE	51	6.95	0.09 (0.03)	4.83	0.03 (0.01)	−0.06	0.2
NYHU	4	0	0.00 (0.00)	0.68	0.00 (0.00)	0.00	0.31
PESU	34	10.5	0.14 (0.04)	0.17	0.00 (0.00)	−0.14	0.00

**Fig. 2:**
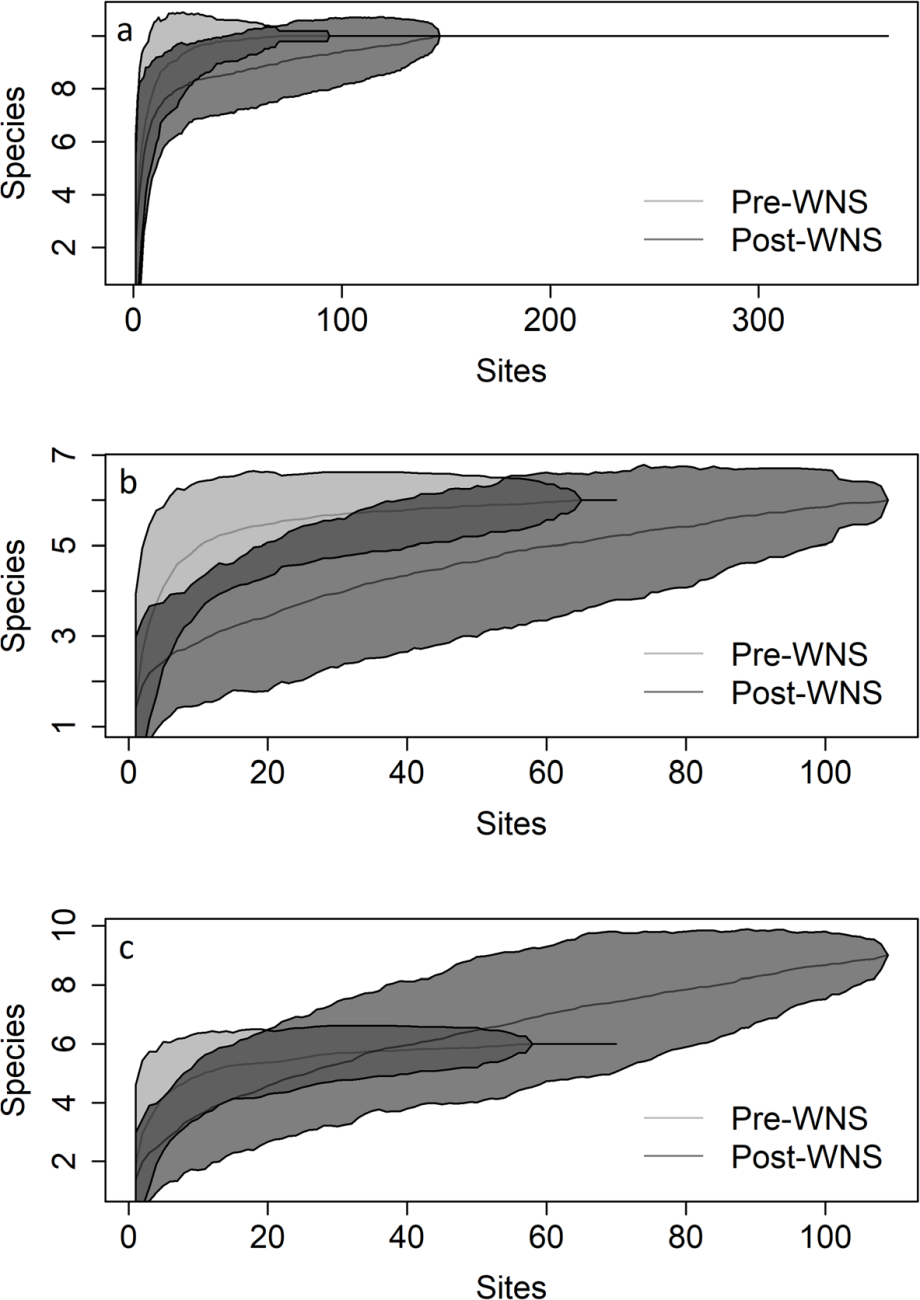
Species accumulation curves for active acoustic and mist netting surveys in the National Park Service National Capital Region 2003–2018. Darker lines and polygons are 2003–2004 samples and standard deviations, and lighter lines and polygons are 2016–2018 samples and standard deviations with **a** acoustic sampling both 2003–2004 and 2017; **b** mist netting with only 6 species captured 2003–2004; and **c** mist netting with all 9 species captured 2003–2004 and 2016–2018

Acoustic species accumulation curves indicated that more effort was required to detect all species post-WNS than pre-WNS, despite more capture success post-WNS. Pre-WNS, 94 active acoustic samples were required to document a species richness of 10 (SD ± 0), whereas post-WNS, the species richness was 9.31 (SD ± 0.68) for the same effort (Fig. 2). Even with the focused post-WNS surveys, pre-WNS netting required 65 netting nights for a species richness of 6 (SD ± 0), but that effort for the same 6 species post-WNS yielded a species richness of 5.07 (SD ± 0.76; Fig. 2). For the post-WNS netting species accumulation curve of all 9 extant species captured, 65 nights yielded a species richness of 7.28 (SD ± 1.20; Fig. 2). All 3 post-WNS species accumulation curves required almost every sample included in the analysis in order to reach the maximum species richness with a standard deviation of 0 (acoustics = 147, both netting = 109).

Based on *t*-tests and overall detections, we observed significant post-WNS increases in *E. fuscus* and significant distribution decreases occurred for *M. lucifugus* and *P. subflavus* (*P* < 0.05; Tables 1 and 3). We also observed significant post-WNS increases in the mean number of captures of *Lasionycteris noctivagans* and decreases in the recorded acoustic calls for *M. leibii* and *M. sodalis* (Tables 1 and 3). Both pre- and post-WNS netting results indicated that species richness was highest in western portions of CHOH, where the NCR is the least urbanized and contains the highest proportions of rural and forested landscapes. However, we did not determine a uniformly-negative trend with regard to urbanization, as we also recorded high species richness in highly-urban units. We captured all *M. septentrionalis* in ROCR, except for a single capture in CATO; 76.9% of *L. noctivagans* in ROCR (*n =* 10); and all but 1 *N. humeralis* in GWMP (Great Falls Park). *Eptesicus fuscus* were active throughout the NCR, with the least activity coinciding with open, agricultural, or suburban portions of CHOH, MONO, and MANA. Most activity was in the D.C. area within the larger contiguous forested portions of ROCR and NACE. We captured more *Lasiurus borealis* west of Great Falls Park (along the geological fall line between the Piedmont and Coastal Plain); however, distribution varied greatly across the landscape.

## Discussion

We determined that active acoustic sampling, as employed in this study, was not suitable for detecting rare bat species on the current post-WNS landscape. Our species accumulation curves reflect decreases in WNS-impacted bat species and higher variation in detections. Similarly, other studies have reported that post-WNS sampling effort should be higher than pre-WNS efforts [46, 62]. The small sampling window for active acoustic methods likely limited detection, especially post-WNS. The limitations of our active acoustic sampling are underscored by not observing post-WNS *M. septentrionalis* echolocation calls in ROCR, whereas our mist-netting results indicated continued presence. There was also a non-significant decline in *M. septentrionalis* echolocation calls across the NCR, despite our capture data indicating a significant decline in post-WNS occurrence. Although our active acoustic sampling techniques may continue to be options for comparing community activity to historical data [46, 63] or for detecting common bats, the sampling period for these methods appears inadequate to fully document post-WNS bat communities.

We recorded significant declines in relative abundance and distribution of WNS-impacted species *M. lucifugus* and *P. subflavus*. These results are similar to those of other summer surveys comparing pre- to post-WNS activity within the northeastern U.S. [21–23]. Though our analyses did not document a significant change in *M. septentrionalis* capture rates, this non-significance is likely the result high post-WNS mist-netting sampling density in ROCR, which may have introduced a overly-conservative bias in our comparison. Nonetheless, we still observed a decrease in distribution throughout the study area.

Increases in post-WNS *E. fuscus* activity in the NCR aligned with documented stable populations in New York summer habitat 2004–2010 [21], 43% increases in a New York hibernaculum 2–3 years after WNS detection [26] and 12% increases in captures approximately 1–3 years after WNS detection in Indiana [29]. *Eptesicus fuscus* populations may have avoided WNS-related declines based on hibernation conditions, e.g., uninfected hibernacula, temperature within hibernacula or shorter hibernation periods, as well as being physiologically less challenged by or resistant to *P. destructans* [24, 25, 64, 65]. *Lasiurus borealis* activity was similar between pre- and post-WNS sampling periods, indicating no expansion into WNS-emptied habitat niches [31] or potential wind-energy driven population declines [66]. Our results are similar to those of pre-WNS versus post-WNS comparisons of bat communities in South Carolina and New York [23, 67].

Whereas urbanization influenced some species’ distributions, it has not appeared to have exacerbated WNS declines. We found our only evidence of recruitment among highly WNS-impacted species in D.C. Some combination of disease resistance, summer habitat, overwintering patterns and a small pre-WNS population of WNS-susceptible species may have led to ROCR being a potential *M. septentrionalis* refugium within the Mid-Atlantic region. Pre-WNS, both *M. septentrionalis* and *M. lucifugus* captures were relatively rare in ROCR compared to most other units, yet *M. septentrionalis* populations appear to be successfully reproducing only within ROCR. Although *M. septentrionalis*’ survival mechanisms are not yet known, remnant *M. lucifugus* populations along the East Coast have exhibited genetically based resistance and avoidance behavior that may reduce WNS impacts [28, 68]. As a mostly-contiguous unit with older-growth, late-successional forest [47], ROCR contained a diverse mix of tree sizes and a high density of large standing snags and trees with cavities and bark conditions conducive to *M. septentrionalis* day-roosting. Given the mature forest, year-round water sources, and surrounding established neighborhoods with numerous tree and anthropogenic day-roost opportunities, ROCR may provide an optimal balance of forest and edge habitat, access to water resources, and both natural and anthropogenic roosting and hibernacula options. Recent overwinter detections of *M. septentrionalis* along coastal areas to the southeast of ROCR [69–72] provide evidence that some individuals are not migrating to *P. destructans*-infected hibernacula to the west in the Appalachian Mountains. The authors’ capture of a *M. septentrionalis* in ROCR on 1 November 2018 provides additional support for this hypothesis. In the more easterly urban areas, we observed slightly higher *E. fuscus* activity overall. Our *E. fuscus* results are similar to those of a landscape-scale survey in Georgia and the Carolinas that reported higher *E. fuscus* abundance within NPS units with high levels of developed land within 5 km [73]. Still, within the D.C. area, we saw higher activity within larger forest patches, similar to observations in Mexico City [38].

We suggest caution regarding the interpretation of pre- and post-WNS comparisons in the NCR and other WNS-affected regions. When comparing post-WNS communities to pre-WNS communities, it is necessary to be cognizant of how improved sampling methods have potentially increased detection and capture rates, whether through compliance with USFWS *M. sodalis* survey guidelines [52] or technological improvements (e.g., passive vs. active acoustics, use of automated identification software, netting equipment). Documented bat echolocation calls and capture rates also may have been influenced by the habitat structure in more urban, fragmented forest stands, with travel corridors concentrating bat activity more than in largely forested landscapes. Moreover, annual weather variation may have influenced differences in acoustic and capture data as well as year-to-year demographic changes [74–76]; 2018 had the most netting samples and had higher levels of precipitation than any other year. The differences in pre- and post-WNS netting species accumulation curves are likely greater than the species accumulation curves demonstrate, as differences in netting effort on each sample night were unaccounted for.

## Conclusions

Based on the documented declines in bat passes and capture rates of *M. lucifugus* and *P. subflavus* populations, these species may be functionally extirpated throughout much of the NCR. Acoustic echolocation calls and netting capture rates for *E. fuscus* increased significantly. Consequently, long-term monitoring would be required in the region to determine *E. fuscus* population increases, and intensive future surveys would be required to assess possible habitat expansion of bats less impacted by WNS (*E. fuscus* and *L. borealis*) into vacant niches. Furthermore, additional assessments in the Mid-Atlantic region are needed to determine whether or how *M. septentrionalis* persistence is linked to atypical overwintering activities, such as use of coastal forests to the east and southeast of the NCR rather than cave hibernacula, high-quality maternity habitat conditions in residual NCR forest patches, or other genetic or behavioral resistance mechanisms.

## Data Availability

The datasets used and/or analyzed during the current study are available from the corresponding author on reasonable request.

## Abbreviations

ANTI: Antietam National Battlefield
CATO: Catoctin Mountain Park
CHOH: Chesapeake and Ohio Canal National Historical Park
D.C.: District of Columbia
GWMP: George Washington Memorial Parkway
HAFE: Harpers Ferry National Historical Park
MANA: Manassas National Battlefield Park
MONO: Monocacy National Battlefield
NAMA: National Mall and Memorial Parks
NACE: National Capital Parks-East
NCR: National Capital Region
NPS: National Park Service
ROCR: Rock Creek Park
WNS: White-nose Syndrome
WOTR: Wolf Trap National Park for the Performing Arts
